# Association of vitamin D status and clinical and radiological outcomes in a treated MS population in Poland

**DOI:** 10.1002/brb3.609

**Published:** 2016-12-07

**Authors:** Sławomir Wawrzyniak, Emilia Mikołajewska, Ewelina Kuczko‐Piekarska, Anna Niezgodzińska‐Maciejek, Aleksander Goch

**Affiliations:** ^1^ Neurology ClinicMilitary Clinical Hospital No. 10 with Policlinic in BydgoszczBydgoszczPoland; ^2^ Department of PhysiotherapyLudwik Rydygier Collegium Medicum in BydgoszczNicolaus Copernicus University in ToruńToruńPoland; ^3^ Neurocognitive LaboratoryCentre for Modern Interdisciplinary TechnologiesNicolaus Copernicus University in ToruńToruńPoland; ^4^ Cardiology and Cardiosurgery ClinicMilitary Clinical Hospital No. 10 with Policlinic in BydgoszczBydgoszczPoland

**Keywords:** immunomodulatory treatment, multiple sclerosis, relapse, relapsing‐remitting, vitamin D

## Abstract

**Background:**

Vitamin D influences the immune system significantly. Previous studies have found that vitamin D deficiency in adolescence can play a significant role in increasing the risk of developing autoimmune diseases including multiple sclerosis. The aim of this study was to investigate the relationship between the vitamin D status in serum and clinical and radiological outcomes in a treated population in Poland.

**Methods:**

Inclusion criteria met 83 adult patients aged 20–61 years with diagnosis of relapsing‐remitting multiple sclerosis, who underwent immunomodulatory treatment which lasted at least 12 months. Levels of serum 25‐hydroxyvitamin D were determined using radio‐immuno assay. Magnetic resonance imaging of the brain and cervical part of a spinal cord was performed each time after 12 months of the treatment. Patients were assessed neurologically after 12 months of treatment, the level of disability was also assessed using Extended Disability Status Scale.

**Results:**

The largest group (63.8%) showed significant vitamin D deficiency (<20 ng/ml), 21.7% showed the suboptimal level of vitamin D (20–30 ng/ml). The normal level of 25(OH)D (>30 ng/ml) was observed in 14.5% of the patients. Statistically significant correlation was observed between the vitamin D status and frequency of relapses.

**Conclusions:**

Our findings confirm that deficiency of vitamin D in patients with MS is correlated with clinical and radiological course of the disease.

## 1 Introduction

Multiple sclerosis (MS) is severe chronic, inflammatory, demyelinating disease of the central nervous system (CNS). There is common belief that complex interplay between various genetic and environmental factors contributes to MS. Vitamin D, aside from regulation of the level of calcium and phosphorus within the human body, influences the immune system significantly. Previous studies have found that vitamin D deficiency in adolescence (as one of the environmental factors) can play a significant role in increasing the risk of developing autoimmune diseases including MS (Fitzgerald et al., 2015; Solomon, 2011).

Studies on proper value of the vitamin D status in the human body have a long history. Subsequent clinical guidelines and recommendations have increased frontier of the normal 25(OH)D levels. According to the current diagnostic criteria formulated by the Polish Interdisciplinary Team (2013), reference values for Central European population are as follows:

– normal level: 30–50 ng/mL (75–125 nmol/L),
– suboptimal level: 20–30 ng/mL (50–75 nmol/L),
– deficiency level: < 20 ng/mL (< 50 nmol/L) (Płudowski et al., 2013).

The aforementioned accurate definition of 25(OH)D levels in patients may be critical while evaluating the efficiency of vitamin D supplementation in remitting‐relapsing MS (RRMS) in clinical trials. Aforementioned definitions are predominantly based on bone calcium metabolism outcomes, since optimal 25(OH)D levels for immune regulation in MS may be higher (Fitzgerald et al., 2015; Munger, Levin, Hollis, Howard, & Ascherio, 2006).

Efforts of many researchers are now focused on the issue of how the vitamin D status can modify the course of MS. Outcomes of the mentioned studies still need for explicit consent (Pouzelo‐Moyano, Benito‐Leon, & Hernández‐Gallego, 2013). Many results indicate an increased risk of MS development in people with low level of 25(OH)D (Alharbi, 2015; Ascherio et al., 2014; Munger et al., 2004, 2006; Simpson et al., 2010) and the correlation between vitamin D status and clinical course of the disease (Rosiak & Zagożdżon, 2012; Runia, Hop, de Rijke, Buljevac, & Hintzen, 2012).

The aim of this study was to investigate the relationship between the vitamin D status in serum and clinical and radiological outcomes in a treated population in Poland.

## 2 Materials and Methods

### 2.1 Ethics statement

The study was conducted in accordance with the Helsinki Declaration and the rules of Good Clinical Practice. Regional Ethical Committee in Bydgoszcz approved the study protocol, and all patients gave written informed consent before trial entry. The secondary analyses presented in this manuscript were conducted anonymously.

### 2.2 Study population

**Figure 1:**
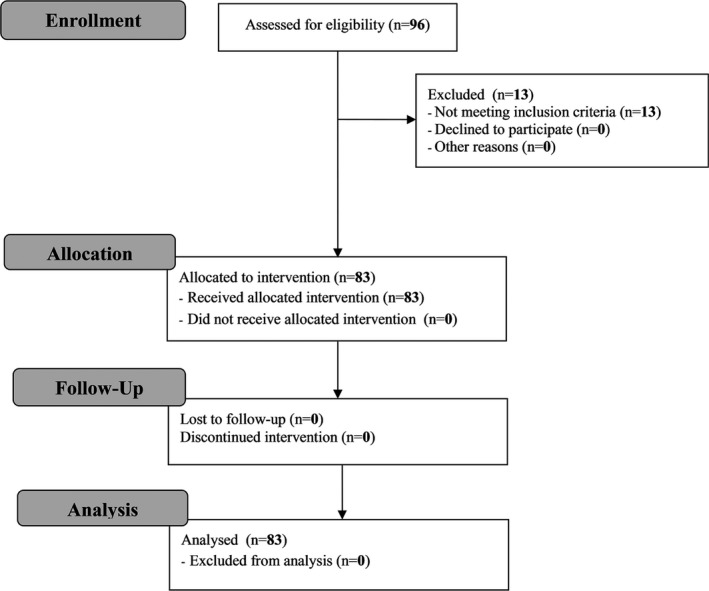
Patients' flow diagram

The study population was determined according to the following criteria. Inclusion criteria result from administrative limitations: all patients who want to take part in the treatment founded by the Polish National Health Fund have to meet inclusion criteria described in regulations (Polish National Health Fund, 2016). Recruitment method was convenience sample. Such recruitment procedure aimed at a sample with sufficient clinical activity to be able to measure associations. Recruitment period was January 2009–January 2014. Figure 1 shows patient's flow diagram.

The research was conducted among 83 adult patients (55 females and 28 males) aged 20–61 years with diagnosis based on the McDonald Criteria, that is, criteria from the International Panel on MS Diagnosis (McDonald et al., 2001): RRMS, who underwent immunomodulatory treatment lasting at least 12 months (2009–2014). Detailed inclusion criteria were as follows:

1. diagnosis of RRMS based on the McDonald Criteria (2010) (McDonald et al., 2001), including Magnetic Resonance Imaging (MRI) before and after the implementation of the contrast;
2. in the case of RRMS: incidence of at least one relapse or at least one new GD+ focus during last 12 months before the qualification to the study;
3. at least 10 points according to the following qualification system:
  - duration of illness:
    – 0–3 years: 6 points,
    – 3–6 years: 4 points,
    – > 6 years: 2 points;
  - number of relapses during last 12 months:
    – ≥ 3: 5 points,
    – 1–2: 4 points,
    – lack of relapses during immunomodulatory treatment (during last 12 months): 3 points,
    – lack of relapses: 1 point;
  - neurological status between relapses (at the beginning of the treatment):
    – EDSS score 0–2: 6 points,
    – EDSS score 2.5–4: 5 points,
    – EDSS score 4.5–5: 2 points (Polish National Health Fund, 2016).

The recruitment procedure mentioned above (inclusion and exclusion criteria) is typical for Polish conditions.

Study location was Bydgoszcz, Poland, and period of recruitment was 2010–2014. Patients were admitted to the immunomodulatory treatment in Neurology Clinic, Military Clinical Hospital No 10. with Polyclinic in Bydgoszcz, Poland. The treatment was provided according to the rules of the drug prescription program of the Polish National Health Fund.

### 2.3 Intervention and assessment

The study design was a prospective before–after study (BAS). Samples were collected at the start of the study and at the end of the study. Patients were recruited at the beginning of the 12‐month period of the treatment, and assessed after the 12 months of the therapy. The immunomodulatory treatment comprised of therapy using interferon beta 1a (INFβ‐1a) (subcutaneous or intramuscular), interferon beta 1b (INF β‐1b), and glatiramer acetate (GA).

Analyses were performed in standard clinical procedures. Levels of serum 25‐hydroxyvitamin D (25(OH)D) in patients with MS were determined using radio‐immuno assay (RIA). Magnetic resonance imaging (MRI) of the brain and cervical part of a spinal cord was performed each time after 12 months of the treatment. New T2 lesions at 12 months were compared to the baseline. MRI was performed using GYROSCAN NT 10 device, 3 mm, sequences T1, T2 FLAIR, contrast agent Magnevist—dose: 0.1 mmol/kg of body weight. T1 sequence shows foci of MS depending on the phase: in the acute phase as a marker than surrounding tissue, and after acute phase: as so‐called “black holes” or similar to the surrounding tissue. Sequence T1 with contrast agent allow for imaging disorders within the brain–blood barrier (darker than surrounding tissue). Aforementioned changes are regarded as the simplest radiological correlations of the MS. Sequence T2 shows both active and nonactive foci of the MS, that is, both acute and long‐term changes, and thus is regarded as the widest applied in the MS imaging.

25(OH)D assay, brand D‐Vitum forte by Oleofarm (liquid protect caps 2000 U) was used for this study.

Patients were assessed neurologically after 12 months of treatment. The level of disability was also assessed using Extended Disability Status Scale (EDSS). Increase ofEDSS > 1 point sustained over 30 days was assessed as the progression of the disease.

Blood samples were collected during fasting in the morning.

### 2.4 Statistical analysis

Where available, descriptive characteristics of study subjects were reported as percentages for categorical variables and mean ± *SD* for continuous variables. Otherwise, median, quartiles, and minimal and maximal values were used. Respectively, parametric and nonparametric tests were used. Fisher's Exact test was used in the analysis of contingency tables. Statistical dependencies between variables were calculated using Spearman's rank correlation coefficient (Spearman's rho). A *p*‐value < 0.05 was considered to be significant. Statistical analysis was performed using the Statistica 10 software.

## 3 Results

The average 25(OH)D level in the study group was 18,6 ng/ml. The largest group (53 patients, i.e., 63.8%) showed significant vitamin D deficiency (<20 ng/ml). Eighteen patients (21.7%) showed the suboptimal level of 25(OH)D (20–30 ng/ml). The normal level 25(OH)D (>30 ng/ml) was observed in 12 patients (14.5%).

All three groups above were similar taking into consideration age, sex, EDSS Outcomes (i.e., level of disability). Exact patient profiles and results are presented in Table 1.

**Table 1:** Patients' overall profile

	25‐OH‐D			
	<20 ng/ml	20‐30 ng/ml	>30 ng/ml	Whole group
*n*	53	18	12	83
%	63.8	21.7	14.5	100
Sex ratio [Female:Male]	2.31:1	1.25:1	2:1	1.96:1
Age [years] (mean ± *SD*)	38.6 ± 9.7	32.5 ± 7.9	36.9 ± 8.3	37.1 ± 9.3
EDSS at admission (mean ± *SD*)	2.2 ± 0.6	1.8 ± 0.4	2.1 ± 0.4	2.0 ± 0.5
Level of the 25‐OH‐D [ng/ml] (mean ± *SD*)	11.9 ± 3.6	23.5 ± 3.5	40.6 ± 11.2	18.6 ± 6.1
Level of the 25‐OH‐D [ng/ml] (median)	10.8	22.8	37.8	15.2
Level of the 25‐OH‐D [ng/ml] First quartile (Q_1_)	7.7	20	30	7.7
Level of the 25‐OH‐D [ng/ml] Third quartile (Q_3_)	19.9	29.9	49.7	49.7

*SD*, Standard Deviation.

Test for difference: ANOVA, *p* < 0.05.

Statistically significant correlation (r = −.30, *p* < .05) was observed between the level of 25(OH)D and frequency of relapses. Relapses were:

– twice as likely in patients with 25(OH)D deficit (43.3%) comparing with patients with suboptimal level (22.2%),
– triple as likely in patients with 25(OH)D deficit comparing with patients with normal level (16.6%, *p* < .05).

Similar results reflecting statistically significant progression of the radiological changes were observed in:

– T2‐weighted scanning sequences (respectively, 28.3% vs. 22.2% vs. 16.6%, *p* < .05),
– contrast‐enhanced examinations (Gd+) (respectively, 15.1% vs. 5.5% vs. 8.3%, *p* < .05).

Only changes reflected in T2‐weighted scanning sequences were statistically significant (r = −.28, *p* < .05).

Statistically significant differences between groups were not observed in EDSS outcomes. Results of the study are presented below (Table 2, Figure 1). We consolidated numbers of events in the suboptimal and normal levels due to statistical reasons (Table 2).

**Table 2:** Outcomes of the treatment depending on the 25(OH)D levels

	25(OH)D		
	<20 ng/ml (*n* = 53)	>20 ng/ml (*n* = 30)	Whole group (*n* = 83)
Relapses during treatment	23 (43.40%)	6 (20.0%)	29 (34.94%)
New changes T2 in MRI	15 (28.30%)	6 (20.0%)	21 (25.30%)
Changes (GD+) in MRI	8 (15.09%)	2 (6.67%)	10 (12.05%)
Durable increase of EDSS>1.0 during treatment	5 (9.43%)	3 (19.4%)	8 (9.64%)

All presented results are dichotomous (Fisher's Exact Test, *p* < .05).

## 4 Discussion

The aim of the study was to investigate the relationship between the level of 25(OH)D in serum and clinical and radiological outcomes in a population treated using the immunomodulatory treatment in multiple sclerosis—there are a few studies concerning this important issue.

The prevalence of MS varies by location on the globe. It increases with latitude (i.e., when traveling from the equator toward northern or southern pole). Despite growing number of publications, the mechanisms influencing the initiation, perpetuation, and therapy of MS remain enigmatic, although it is established that a combination of genetic predisposition and environmental stimulation is required (Nedeljkovic et al., 2016; Selmi, Papini, Pugliese, Alcaro, & Gershwin, 2011). Thus, true may be an assumption that vitamin D deficiency can constitute important element among causative agents of the MS, and it may explain such altered MS incidence (Ascherio et al., 2014).

Paul Goldberg is the first author who mentioned the hypothesis linking a deficiency of vitamin D and MS prevalence (1974). He studied the relationship between environmental determinants (such as sunlight, dietary factors, etc.) and MS prevalence (Goldberg, 1974). Further studies have confirmed this relationship (Alonso & Hernán, 2008; Embry, Snowdon, & Vieth, 2000; Simpson, Blizzard, Otahal, Van der Mei, & Taylor, 2011). This situation prompted scientists and clinicians to further research the influence of vitamin D and its supplementation to both the natural course of the disease (Soilu‐Hänninen et al., 2005) and effectiveness of the treatment (Løken‐Amsrud et al., 2012; Soilu‐Hänninen et al., 2012; Stein et al., 2011).

Low level of 25(OH)D is more often observed in patients with MS than in general population (Ascherio, Mungen, & Simon, 2010; McDowell et al., 2011; Munger et al., 2004). The normal level of 25(OH)D was observed in 14.5% of patients with MS. A significant deficiency of 25(OH)D was observed in 63.8% of patients with MS—they require pharmacological supplementation (Ascherio et al., 2014). Subtherapeutic level of 25(OH)D was observed in 21.7% patients with MS.

The clinical efficacy of vitamin D in patients with MS is not clearly defined so far. Some of the previous research showed a correlation between seasonal fluctuations of 25(OH)D level, the frequency of relapses, and the number of demyelinating lesions observed during contrast‐enhanced examinations (MRI) (Embry et al., 2000; Tremlett et al., 2008). There is evidence that level of the vitamin D was significantly lower in patients with relapse in the course of the MS. There is a lack of consistency among the findings of researchers as to whether relapse is a cause or effect of the vitamin D deficit (Soilu‐Hänninen et al., 2005).

Prospective research by Simpson et al. (*n* = 145) showed an association between 25(OH)D level and frequency of relapses. Moreover, each 10 nmol/L increase of 25(OH)D level results in up to a 12% reduction in risk of relapse (Simpson et al., 2010). Our results are consistent with findings above. We observed increased frequency of relapses in patients with 25(OH)D deficit <20 ng/ml, compared to patients with a normal 25(OH)D level (>30 ng/ml) (respectively, 43.3% vs. 16.6%, *p* < .05). There were observed similar correlations concerning radiological changes (MRI).

Different results were presented by Løken‐Amsrud et al., 2012. They analyzed outcomes in 88 patients with MS, 6 months before and 18 months after initiation of IFN‐β treatment. The only observed change was a decrease in the number of demyelinating lesions in patients with higher 25(OH)D level.

A systematic review of randomized, double‐blind, placebo‐controlled trials performed by Pozuelo‐Moyano et al. showed that there is a lack of evidence concerning vitamin D as a treatment for MS. There are only a few large, well‐designed randomized trials concerning the issue above. There is a need for further research and detailed analyses aiming at definite solving important scientific and clinical problem concerning the influence of vitamin D on the course of MS (Munger et al., 2006).

A recent study by Munger et al., 2014 provided evidence that an unbalanced 25(OH)D gene expression system may affect MS activity. Also, a study by Waschbisch et al., 2014 provided evidence that IFN‐mediated induction of ILT3 can be potentiated by vitamin D. Results of study by Rinaldi et al., 2015 confirmed that MS patients have higher vitamin D‐binding protein (DBP) levels than healthy subjects.

On the other hand, a recent study Røsjø et al., 2015 showed results of MRI and inflammation markers across patients categorized by mean vitamin D values, suggesting a lack of vitamin D influence on interferon‐β1a treatment effects.

The main limitation of this study is a relatively small sample and lack of control group. Number of patients (*n* = 83) is similar to the lower values from the previous studies—it varied from *n* = 88 (study by Røsjø et al., 2015) to *n* = 465 (study by Munger et al., 2014) and may be regarded as proper for preliminary findings. Aforementioned number of patients seems to be appropriate for main aim of the study: investigation of the relationship between the 25(OH)D levels in serum and course of the disease. Thus, sample size and therefore the robustness of the data are not questionable. Another study limitation is seasonal fluctuation. Our findings can be biased by a season‐effect since deseasonalized 25(OH)D levels were not provided. Clear reference values confirmed by guidelines allow avoiding the other limitation. Differences in the biological effects of vitamin D metabolites were previously described (Correale, Ysrraelit, & Gaitan, 2010). These outcomes need further research. We also consolidated number of events in the suboptimal and normal levels in Table 2 due to small numbers. Another limitation was the various vitamin D assays used.

There may be assumption that the distribution of immune‐modulating treatments (IFNb+GA) between all treatment groups influences results. But article by McGraw and Lublin showed lack of statistically relevant differences between IFNb, GA, and INFb+GA (McGraw & Lublin, 2013).

Assumption that disease activity generally improved with higher 25(OH)D levels may also be true, but effect modification by treatment class is still discussed and needs additional research, for example, no significant associations were found for relapses (Rotstein et al., 2015). Thus, aforementioned analysis may diminish results of the study. Study by Rotstein et al., 2015 is relatively new and is still discussed (Konieczka, Koch, Binggeli, Schoetzau, & Kesselring, 2016; Polivka, Polivka, Krakorova, Peterka, & Topolcan, 2016).

We took into consideration diverse disease‐modifying treatments (DMTs) but we excluded many of them such as treatment for secondary disease or simultaneous rehabilitation. Patients in different vitD strata were assessed as comparable regarding baseline MRI and DMTs.

Evidence above supports the hypothesis that 25(OH)D levels (e.g., through supplementation) may influence the course of the therapy in MS patients treated with IFN‐beta‐1b; but there is still a lack of common agreement. We have high hopes for a settlement to this dispute during our further studies. To avoid aforementioned limitations, directions of our further studies will cover monitoring and managing 25(OH)D levels in early MS patients treated with IFN‐beta‐1b, and also investigating the biological mechanisms underlying aforementioned observations.

To sum up, vitamin D insufficiency is regarded as a potential risk factor for MS, associated with a higher disease activity. Based on the current knowledge, we cannot anticipate whether vitamin D bioavailability is associated with RRMS patients. Results of previous studies addressing effects of oral high‐dose vitamin D are not consistent; there is a need for larger studies such as SOLAR. But we believe that our outcomes add an important element to explaining the vitamin D role in MS patients.

## 5 Conclusions

Our findings confirm that deficiency of vitamin D in patients with MS is correlated with clinical and radiological course of the disease. Further research, including studies comparing the effectiveness of the immunomodulatory treatment in MS after vitamin D supplementation, can constitute an important tool to optimizing the treatment in patients with MS.

## Conflict of Interest

None declared.
